# Radiological profile of patients undergoing Chest X-ray and computed tomography scans during COVID-19 outbreak

**DOI:** 10.12669/pjms.37.5.4290

**Published:** 2021

**Authors:** Sohail Ahmed Khan, Murli Manohar, Maria Khan, Samita Asad, Syed Omair Adil

**Affiliations:** 1 Sohail Ahmed Khan, Assistant Professor, Dow Institute of Radiology, Dow University of Health Sciences, Karachi, Pakistan; 2 Murli Manohar, Instructor, Dow Institute of Radiology, Dow University of Health Sciences, Karachi, Pakistan; 3 Maria Khan, Instructor, Dow Institute of Radiology, Dow University of Health Sciences, Karachi, Pakistan; 4 Samita Asad, Resident, Dow Institute of Radiology, Dow University of Health Sciences, Karachi, Pakistan; 5 Syed Omair Adil, Lecturer Biostatistics, School of Public Health, Dow University of Health Sciences, Karachi, Pakistan

**Keywords:** COVID-19, Computed tomography, X-rays, Chest

## Abstract

**Background & Objective:**

Radiology has played a significant role in the diagnosis and quantifying the severity of COVID 19 pulmonary disease. This study was conducted to assess patterns and severity of COVID-19 pulmonary disease based on radiological imaging.

**Methods:**

A prospective observational study was conducted in a large tertiary care public sector teaching hospital of Karachi, Pakistan from June 2020 till August 2020. All confirmed and suspected COVID-19 patients referred for chest X-rays and computed tomography (CT) scans were evaluated along with RT-PCR results. Suspected patients were followed for RT-PCR. Radiological features and severity of imaging studies were determined.

**Results:**

Of 533 patients in whom X-rays were performed, majority had severe/critical findings, i.e., 304 (57.03%). Of 97 patients in whom CT scan was performed, mild/moderate findings were observed in 63 (64.94%) patients. Of 472 patients with abnormal X-rays, majority presented with alveolar pattern 459 (97.2%), bilateral lung involvement 453 (89.6%), and consolidation 356 (75.4%). Moreover, lobar predominance showed lower zone preponderance in 446 (94.5%) patients. Of 88 patients with abnormal CT findings, ground-glass opacity (GGO) 87 (98.9%) and crazy paving 69 (78.4%) were the most common findings. An insignificantly higher association of PCR positive cases was observed with severe/critical X-rays (p-value 0.076) and CT scan findings (p-value 0.431).

**Conclusion:**

Most common patterns on CT scans were GGO and crazy paving. While on chest radiographs, bilateral lung involvement with alveolar pattern and consolidation were most common findings. On X-rays, majority had severe/critical whereas CT scan had mild/moderate findings.

## INTRODUCTION

COVID-19 pandemic has been devastating throughout the world and remains a challenge in terms of diagnosis and management.^1^-^4^ Although the current diagnostic criterion for COVID-19 is the real-time reverse transcription-polymerase chain reaction (RT-PCR),^5^ radiology has also played a significant role in the diagnosis and quantifying the severity of COVID 19 pulmonary disease throughout the world.^6^,^7^ As per guidelines by the Radiological Society of North America (RSNA), imaging is only indicated in positive COVID-19 patients having worsening respiratory status and in suspected COVID-19 patients having moderate to severe symptoms with the unavailability of RT-PCR tests.^8^

X-rays and Computed Tomography (CT) became more prevalent globally in helping to increase awareness and track the progress of Covid-19 pulmonary disease.^9^-^11^ The disease profile evolved rapidly, as evident by literature, a spectrum of significant imaging findings was noted in asymptomatic patients and, on the other hand, critically ill patients with no significant radiological manifestations were seen.^12^,^13^ However, it has been noted that chest X-ray is less sensitive in detecting the early manifestations of pulmonary disease, although it can detect the disease in advanced stages. CT scan, on the other hand, can detect early parenchymal lung disease, disease progression, and alternative diagnoses.^14^ Due to the relative constraints of CT scanner availability, higher radiation dose, and decontamination procedure following imaging, patients are not routinely referred for CT scan.^15^ Chest X-ray is the initial imaging investigation in patients with respiratory symptoms; yet, much less has been written about it for COVID-19.^16^

This study was conducted to identify the role of radiological imaging in the assessment of disease severity in a cohort of the Pakistani population as no published study of such a large sample size was detected during data search for both CT and X-ray.

## METHODS

This prospective observational study has been conducted at the Dow institute of Radiology, Dow University of Health Sciences from June 2020 to August 2020. Approval of the Ethical Review Board (Ref: IRB-1694/DUHS/Approval/2020, dated: 27^th^ June 2020) was obtained; informed consent from all patients undergoing chest X-ray and CT scans was taken regarding the publication of their data while maintaining confidentiality.

Epi Info sample size calculator is used for the estimation of sample size taking confidence interval 99.9%, margin of error 5%, reported frequency of ground-glass opacities on CT lung 86%.^17^ The estimated sample size came out to be 319. However, a total of 596 patients were included, out of which 499 had a chest X-ray only, 63 had a chest CT scan only, and 34 patients had both chest X-ray and CT scans. Chest X-ray of all the suspected COVID-19 patients (having fever > 38°C with symptoms of lower respiratory tract illness like a cough or shortness of breath and history of traveling from abroad or contact with a RT-PCR positive COVID-19 patient within 14 days of onset of symptoms or with fever >38°C with a severe acute respiratory illness like pneumonia or acute respiratory distress syndrome requiring hospitalization along with confirmed cases (RT-PCR positive) admitted in hospital isolation wards and intensive care units were obtained through portable X-ray machines. Both non-contrast imaging after volumetric like high resolution CT and contrast-enhanced CT chest were performed as per the primary physician’s request. All X-rays and CT scans were done after using personal protective equipment and following the guidelines for safe exposure to limit cross-infection.

### Image Analysis

All cases were reported by two junior radiologists and two senior radiologists having more than 10 years of reporting experience. The presence of imaging features including consolidation, air space shadowing, and pleural effusions were noted in X-rays of all suspected and confirmed patients. CT images were classified predominantly as having ground-glass opacities, consolidations, cavitation, nodular opacities, crazy paving, pleural/pericardial effusions, and lymphadenopathy. Findings were further categorized into the zonal (X-ray) and lobar predominance (CT).

### Radiological Scoring:

### Chest X-ray severity score

Severity was measured through Radiographic Assessment of Lung Edema (RALE) criteria that includes no involvement, mild (<25%) involvement, moderate (25-50%) involvement, severe (50-75%) involvement, critical (>75%) involvement.^18^

### CT scan chest severity score

Involvement of 0% lung was considered as none, 1-25% was considered as mild, 26-50% was considered as moderate, 51-75% was considered as severe, and 76-100% was considered as critical.^19^ X-ray and CT severity were further subcategorized into two groups: mild and moderate cases were merged and labeled as a minor group (having <50% involvement). While severe and critical cases were merged and labeled as a major group (>50% involvement). A detailed clinical history was obtained from patients through a pre-structured questionnaire. The epidemiological and clinical data including age, sex, traveling history, history of contact with RT-PCR positive patient, clinical symptoms including fever, cough, sputum, shortness of breath, diarrhea, body pain and chest pain, duration of symptoms, and comorbidities including hypertension, diabetes and chronic obstructive pulmonary disease was recorded. RT-PCR of all the suspected radiological cases was followed, and the cases were finally grouped as positive or negative for COVID-19.

### Statistical Analysis

SPSS version 21 was used for analysis. The mean ±SD for age and onset days of COVID-19 symptoms was determined. Frequency and percentages were calculated for gender, history of travel, history of contact with a COVID-19 patient, symptoms, past medical history, CT chest, and X-ray findings. A comparative analysis of groups of X-ray and CT severity with patients’ demographic data and clinical characteristics and RT-PCR result was done. Inferential statistics were explored using Independent t-test, and Chi-square/Fisher-Exact test applied. p-value ≤0.05 was considered as significant. Diagnostic accuracy of X-ray and CT scan was also calculated using PCR as gold standard.

## RESULTS

Of 596 patients, the mean age was 54.58 ±13.64 years. There were 414 (69.5%) males and 182 (30.5%) females. The mean onset of symptoms was 3.91 ±1.41 days. The cough was the most predominant symptoms observed in 544 (91.3%), fever in 473 (79.4%), shortness of breath in 442 (74.2%), and body pain in 405 (67.9%) patients. The frequency of comorbidities showed that hypertension was observed in 49 (8.2%), diabetes in 27 (4.5%), and COPD in 16 (2.7%) patients.

**Fig.1:**
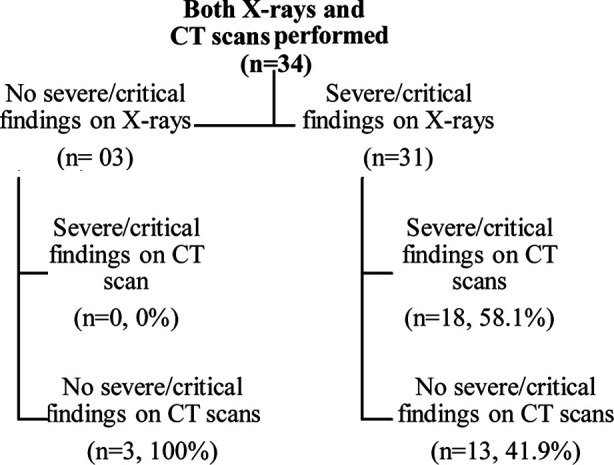
Severity findings in patients with both X-ray and CT examinations (n=34).

X-ray findings were reported in 533 patients. Of these, normal X-ray findings were observed in 61 (11.4%), mild in 58 (10.9%), moderate in 110 (20.6%), severe in 117 (19.6%), and critical in 187 (35.1%) patients. (Fig.1) Comparative analysis of severe/critical findings on X-rays with demographic and clinical characteristics showed a significantly higher proportion of X-ray severity in patients with shortness of breath (p-value <0.001) and chest pain (p-value 0.002). (Table-I)

**Table-I:** Comparative analysis of X-ray and CT severity findings with demographic and clinical characteristics of the patients.

	Severe/critical findings on X-ray (n=533)			Severe/critical findings on CT (n=97)			Severe/critical findings on both X-ray & CT (n=34)		

	Yes (n=304)	No (n=229)	p-value	Yes (n=30)	No (n=67)	p-value	Yes (n=18)	No (n=16)	p-value
***Age, years***	55.01 ±13.05	54.28 ±14.48	0.543^€^	58.33 ±10.14	54.72 ±13.96	0.205^€^			
***Gender***									
Male	211 (69.4)	157 (68.6)	0.834^¥^	21 (70)	48 (71.6)	0.869^¥*^	12 (66.7)	11 (68.8)	0.897
Female	93 (30.6)	72 (31.4)		9 (30)	19 (28.4)		6 (33.3)	5 (31.3)	
***The onset of symptoms, days***	4.21 ±1.33	4.07 ±1.26	0.217^€^	3.30 ±1.77	2.40 ±1.06	0.003^€^			
***Symptoms***									
Fever	211 (69)	175 (76.4)	0.031^¥^	21 (70)	52 (77.6)	0.422^¥^	16 (88.9)	14 (87.5)	0.900
Shortness of breath	254 (83.6)	141 (61.6)	<0.001^¥*^	23 (76.7)	53 (79.1)	0.788^¥*^	16 (88.9)	13 (81.3)	0.530
Cough	287 (94.4)	213 (93)	0.508^¥^	22 (73.3)	54 (80.6)	0.422^¥^	17 (94.4)	15 (93.8)	0.932
Sputum	20 (6.6)	8 (3.5)	0.114^¥^	13 (43.3)	42 (62.7)	0.075^¥^	3 (16.7)	4 (25)	0.549
Diarrhea	12 (3.9)	16 (7)	0.119^¥^	1 (3.3)	2 (3)	0.927^¥^	1 (5.6)	0 (0)	0.339
Chest Pain	246 (80.9)	159 (69.4)	0.002^¥*^	19 (63.3)	37 (55.2)	0.455^¥^	14 (77.8)	16 (100)	0.045
Body Pain	238 (78.3)	164 (71.6)	0.076^¥^	15 (50)	16 (23.9)	0.011^¥^	14 (77.8)	14 (87.5)	0.458
***Comorbidity***									
HTN	15 (4.9)	19 (8.3)	0.116^¥^	6 (20)	12 (17.9)	0.807^¥^	2 (11.1)	1 (6.3)	0.618
Diabetes	5 (1.6)	10 (4.4)	0.060^¥^	2 (6.7)	12 (17.9)	0.145^¥^	0 (0)	2 (12.5)	0.122
COPD	5 (1.6)	6 (2.6)	0.433^¥^	1 (3.3)	4 (6)	0.587^¥^	18 (100)	16 (100)	-
***Travel History***	15 (4.9)	12 (5.2)	0.873^¥^	4 (13.3)	5 (7.5)	0.042^¥*^	2 (11.1)	1 (6.3)	0.618
***Contact History***	66 (21.7)	41 (17.9)	0.277^¥^	15 (50)	43 (64.2)	0.188^¥^	5 (27.8)	4 (25)	0.855

€ Independent t-test applied,

¥ Chi-square/Fisher-Exact test applied,

* p-value ≤0.05

CT scans were performed in 97 patients. Of these normal CT findings were observed in 9 (9.3%), mild in 27 (27.8%), moderate in 36 (37.1%), severe in 20 (20.6%), and critical in 5 (5.2%) patients. Comparative analysis of severe/critical findings on CT scans with demographic and clinical characteristics showed a significantly higher proportion of CT severity in patients with longer duration of symptoms (p-value 0.003), patients with the complaint of body pain (p-value 0.011), and travel history (p-value 0.042). (Table-I)

There were 34 (5.70%) patients in whom both X-ray and CT examinations were performed. Of these 34 patients, 31 (91.2%) patients showed severe/critical findings on X-rays, and 3 (8.82%) showed no severe/critical findings on X-rays. Of 31 patients in whom X-ray findings showed severe/critical findings, only 18 (58.1%) were found severe/critical on CT scans whereas in 3 patients in whom X-ray findings showed no severe/critical findings, CT scans also showed no severe/critical findings in all these patients, i.e. 3 (100%). (Fig.1)

Radiological profile of abnormal X-ray findings showed that of 472 patients in whom abnormality was observed, the majority of the patients were presented with an alveolar pattern, bilateral lung involvement, and consolidation, i.e. 459 (97.2%), 453 (89.6%), and 356 (75.4%) respectively. Moreover, lobar predominance showed lower zone preponderance in 446 (94.5%) patients. CT findings showed that out of 88 patients in whom abnormality was observed, ground-glass opacity (GGO), >50% GGO, and crazy paving were observed in most of the patients, i.e. 87 (98.9%), 68 (77.3%), and 69 (78.4%) respectively. (Table-II)

**Table-II:** Radiological Profile of abnormal X-ray and CT findings.

	n	%
***X-ray Radiological Profile (n=472)***		
***Lung Findings***		
Alveolar Pattern	459	97.2
Consolidation	356	75.4
Bilateral lung involvement	453	89.6
Pleural Effusion	14	3
***Lobar Predominance***		
Upper Zone	1	0.2
Lower Zone	446	94.5
Both	17	3.6
***CT Radiological Profile (n=88)***		
Lobar Predominance		
Right Upper Lobe	76	86.4
Right Middle Lobe	63	71.6
Right Lower Lobe	86	97.7
Left Upper Lobe	74	84.1
Left Lower Lobe	86	97.7
***Predominant Distribution***		
Peripheral	70	79.5
Perihilar	2	2.3
No Predominance	16	18.2
***CT Findings***		
Ground Glass Opacity (GGO)	87	98.9
Consolidation	48	54.5
>50% GGO	68	77.3
>50% Consolidation	11	12.5
Crazy paving	69	78.4
Cavitation	0	0
Nodules	4	4.5
Pleural Effusion	4	4.5
Pericardial Effusion	0	0
Enlarged Nodes	1	1.1

Diagnostic accuracy of X-ray showed sensitivity, specificity, PPV, and NPV as 88.87%, 66.67%, 99.79%, and 3.28% respectively. Similarly, diagnostic accuracy of CT scan was found to be 96.67%, 85.71%, 98.86%, and 66.67% respectively. (Table-III)

**Table-III:** Diagnostic accuracy of X-ray and CT scans taking PCR findings as gold standard.

	PCR Finding						
Abnormal X-ray	Yes	No	Sensitivity	Specificity	PPV	NPV	Overall Diagnostic Accuracy
Yes	471	1	88.87%	66.67%	99.79%	3.28%	88.74%
No	59	2					
***Abnormal CT***							
Yes	577	10	96.67%	85.71%	98.86%	66.67%	95.88%
No	9	0					

X-ray findings showed an insignificantly higher proportion of PCR positive cases in whom severe/critical findings on X-rays were present as compared to those in whom severe/critical findings on X-rays severity were not present, i.e. 304 (100%) and 226 (98.7%) respectively, p-value 0.076. Similarly, CT findings showed an insignificantly higher proportion of PCR positive cases in whom severity was present as compared to those in which severity was not present, i.e. 29 (96.7%) and 61 (91.0%) respectively, p-value 0.431.

## DISCUSSION

**Fig.2:**
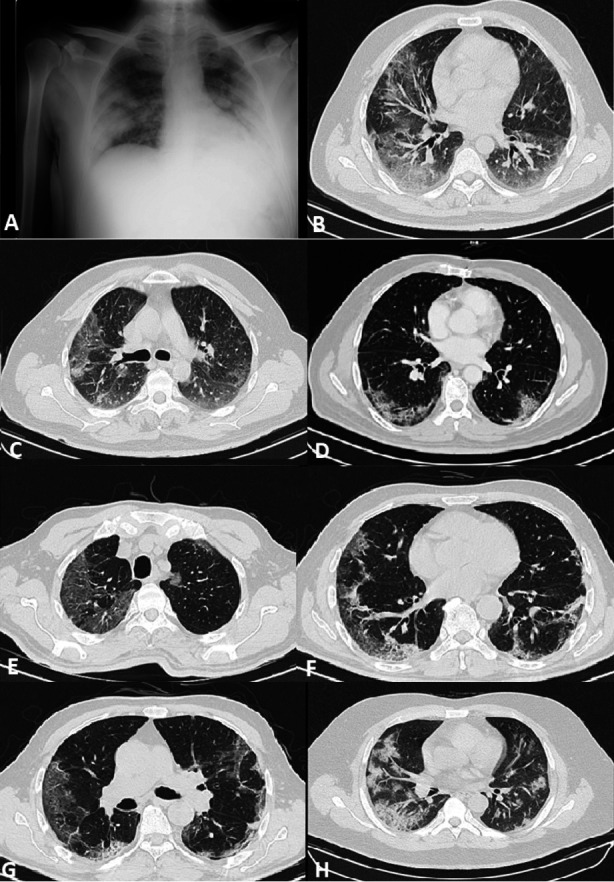
(A) Chest X-ray supine view of a 45 years male patient with severe dyspnea showing bilateral mid and lower zone airspace shadowing with peripheral predilection. (B) HRCT chest lung window axial images of same patient showing bilateral peripheral subpleural areas of ground glass haze with lower lobe predominance. (C,D) CT chest contrast axial image in lung window showing subpleural ground glass haze, reticulation and crazy paving in lower lobes in a 65 years male patients with fever, cough and sore throat. (E-G) HRCT chest lung window axial images of a 60 years male patient with fever, cough, dyspnea and body aches showing bilateral ground glass haze and crazy paving in peripheral subpleural location of both lungs. (H) HRCT chest lund window image of a 48 years male patient with fever, dyspnea and body ache showing bilateral peripheral subpleural consolidations with minimal ground glass haze in both lungs.

This study was conducted in a large public sector tertiary care hospital specified by the government for COVID-19 with designated three isolation wards and three ICUs. Moreover, in this hospital, investigations for the COVID-19 patients were offered free of cost, therefore we were able to cater the needs of a large population. The findings of the study identified the occurrence of ground-glass opacity as the most common finding in CT scans of COVID-19 pulmonary disease. (Fig.2) This is coinciding with the outcomes of previous studies done in China, America, Europe, and Pakistan.^20^-^23^ It is present in almost all abnormal CT scans making it an essential diagnostic feature. A study by Li, Y. and Xia, L confirmed the absence of ground-glass opacity in COVID 19 positive patients to be a rare occurrence.^24^ Crazy paving was the second most common finding, in contrast to a much lower reported frequency in the previously published studies in China, Italy, and also in other cities of Pakistan.^25^-^27^ A similar high frequency was reported by Li K et al., who compared the chest CT features associated with severe and critical COVID-19 pulmonary disease with mild cases and stated that crazy paving is associated with a higher severity of disease and another study suggested that it could be used as a marker for disease progression.^28^ Therefore, the other studies which showed lower frequency could be assumed to be conducted in a less severely affected population and with smaller sample size. Consolidation was the next most common finding when it was compared with ground-glass opacity, more than half of the scans had ground-glass opacity. All these features were most commonly seen bilaterally in the subpleural peripheral location of the lower lobes, synchronizing with the findings of various studies.^16^,^29^ In this study, pleural effusion, lymphadenopathy, cavitation, and pericardial effusion were rarely noted, as was seen in previous literature. In this context, Bai and his colleagues have concluded that these particular findings were found to be more prevalent in viral pneumonia other than COVID-19 pulmonary disease.^30^

In terms of severity, most of the patients who presented for CT scan showed moderate severity, and the patients who presented for X-rays showed critical severity. There could be few reasons for this; firstly, the utility of CT scan as a screening tool was discouraged by leading radiological societies.^8^ and was indicated in moderate to severe cases only as mentioned previously. Secondly, portable X-rays, as opposed to CT, were more commonly being carried out in severely ill patients admitted in ICUs (including those on mechanical ventilation). Due to the constraints of logistics of shifting to the radiology department and rigorous time-consuming decontamination measures that followed. This may have had an impact on results.

Regarding the PCR negative patients undergoing the X-ray, none were present in the major group. However, more than half were found to be in the major group on the CT scan, suggesting that the CT scan is highly sensitive for the detection of disease in the presence of negative PCR.^31^ This can be attributed to the fact that PCR has a high false-negative rate, and the unavailability of testing kits in early outbreak restricted the prompt diagnosis of infected patients. Therefore, the role of the CT scan was recognized as a diagnostic tool. In a low resource country like Pakistan, CT scan was mostly advised by physicians in highly suspected patients. On the other hand, for severely ill and confirmed cases, the X-rays were used as a diagnostic tool due to cost-effectiveness and logistic problems, as discussed earlier.

### Strength and Limitations of the study

The findings of this study can be highlighted in the light of certain limitations. Firstly, in the current study, follow-up of patients was not carried out. Therefore, disease outcome was not assessed. Secondly, the patients were not categorized into groups according to the duration of symptoms to study the various imaging stages of this disease as present in previous literature.^32^-^34^ Moreover, some patients may have received therapy in the form of antimicrobial drugs or steroids, which may have altered the disease severity at the time of imaging and this factor was not taken into account. The strength of this study was a larger sample size including both X-ray and CT scans. To the best of our knowledge no such study in Pakistan has been conducted so far that has included both radiological modalities, evaluating data for COVID-19 pulmonary disease for this large number of patients. Further studies are recommended to evaluate the disease pattern over time with the help of follow-up imaging to determine patient outcomes.

## CONCLUSION

In our cohort, most of the patients undergoing chest X-ray showed severe lung involvement, whereas most of the other patients undergoing CT scan chest revealed mild to moderate lung disease.

### Abbreviations:

**COVID-19** Coronavirus disease 2019

**CT** Computed Tomography

**GGO** Ground-Glass Opacity

**RALE** Radiographic Assessment of Lung Edema

**RSNA** Radiological Society of North America

**RT PCR** Reverse Transcriptase – Polymerase Chain Reaction

### Authors’ Contributions:

***SAK, SOA, MM, MK:*** Conception and design of the study.

***SOA:*** Data collection, analysis, and interpretation.

***SA, MK, MM:*** Manuscript writing.

***SAK:*** Critical Review and final approval of the manuscript.

***SOA:*** Responsible and accountable for the accuracy or integrity of the work.
